# Cetuximab and IL-15 Promote NK and Dendritic Cell Activation In Vitro in Triple Negative Breast Cancer

**DOI:** 10.3390/cells9071573

**Published:** 2020-06-28

**Authors:** Estefanía Paula Juliá, José Mordoh, Estrella Mariel Levy

**Affiliations:** 1 Centro de Investigaciones Oncológicas CIO-FUCA, Ciudad Autónoma de Buenos Aires C1426AOE, Argentina; estefaniajulia@hotmail.com (E.P.J.); jmordoh@alexanderfleming.org (J.M.); 2 Fundación Instituto Leloir, Ciudad Autónoma de Buenos Aires C1405BWE, Argentina; 3 Instituto Alexander Fleming, Ciudad Autónoma de Buenos Aires C1426AOE, Argentina

**Keywords:** NK cells, dendritic cells, ADCC, Cetuximab, triple negative breast cancer

## Abstract

Triple Negative Breast Cancer (TNBC) treatment is still challenging, and immunotherapy is a potential approach in this tumor subtype. Cetuximab is an IgG1 monoclonal antibody (mAb) directed against Epidermic Growth Factor Receptor (EGFR), a protein overexpressed in a subgroup of TNBC patients and associated with poor prognosis. Previously, we demonstrated in vitro that Cetuximab triggers Ab-dependent cell cytotoxicity against TNBC cells. In this study, using co-cultures including TNBC cells, and NK and Dendritic Cells (DCs) from healthy donors, we studied the effect of Cetuximab-activated NK cells on DC function. Given that we already demonstrated that TNBC has an immunosuppressive effect on NK cells, we also tested Cetuximab combination with IL-15. We determined that Cetuximab opsonization of TNBC cells increased IFN-γ and TNF-α production by NK cells co-cultured with DCs. Moreover, we showed that NK cells activated by TNBC cells opsonized with Cetuximab promoted tumor material uptake and maturation of DCs, as well as their ability to produce IL-12. Furthermore, the stimulation with IL-15 increased the activation of NK cells and the maturation of DCs. These results suggest that IL-15 may enhance the efficacy of Cetuximab in the treatment of TNBC by promoting activation of both NK cells and DCs.

## 1. Introduction

Triple Negative Breast Cancer (TNBC) is an aggressive subtype of breast cancer (BC) associated with poor outcomes that lack the expression of hormone receptors and ERBB2 [1]. The standard treatment for TNBC patients is chemotherapy, but it is limited since the duration of response is frequently short, and rapid relapses are common [2]. TNBC exhibits a higher amount of tumor-infiltrating lymphocytes (TILs) relative to other breast cancer subtypes [3]. Furthermore, TIL count is associated with improved survival, reduced recurrence risk, and augmented probability of response to neoadjuvant chemotherapy in early-stage TNBC [4]. These and other characteristics make TNBC patients proper candidates for immunotherapy [5].

NK cells are effectors of innate immunity with the ability to induce the death of tumor cells and virus-infected cells in the absence of specific immunization [6]. The recognition of ligands specifically expressed by malignant and stressed cells without previous sensitization is a hallmark of NK lymphocytes [7]. Moreover, NK cells recognize cells with MHC-I downmodulation, a common mechanism by which cancer cells avoid T cell recognition [8]. Besides their killing capacity, NK cells are recognized as major producers of IFN-γ in many physiological and pathological conditions. They also produce an array of other cytokines, both proinflammatory and immunosuppressive, and growth factors. Proinflammatory cytokines from activated NK cells provide dendritic cells (DCs) signals for their activation, maturation and cytokine production, thereby promoting adaptive immunity [9]. Reciprocally, DCs can stimulate NK cells by soluble as well as contact-dependent activators, thereby enhancing their cytokine production, proliferation, survival, and cytotoxicity [10]. This DC–NK cell crosstalk would take place, in vivo, in the lymph nodes [11,12] and inflammation sites, at peripheral tissues such as the skin and mucosa [13] and solid tumor microenvironments [14].

In the last few years, our group has studied NK lymphocytes as potential effectors of the immune response against BC. Peripheral blood NK cells from early-stage BC patients were phenotypically similar to those from healthy donors [15]. Nonetheless, BC cell lines exposed to patient’s peripheral lymphocytes were resistant to NK cell-mediated lysis. Direct killing against TNBC cells may be limited by their high expression of ligands of NK cell inhibitory receptors, such as MHC-I [16]. To overcome this resistance, we took advantage of a key mechanism that allows NK cells to increase their lytic capacity, using a therapeutic antibody that triggers Ab-dependent cell cytotoxicity (ADCC). Thus, we utilized Cetuximab, an IgG1 monoclonal antibody (mAb) directed against Epidermic Growth Factor Receptor (EGFR), an antigen overexpressed in a subgroup of TNBC patients and associated with poor prognosis [15,17,18]. Cetuximab is thoroughly used in clinical practice in colorectal, lung, and head and neck cancer [19,20,21] and has been evaluated for TNBC treatment [22]. Previously, we demonstrated in vitro that the activation of NK cells from BC patients through Fcγ receptor resulted in the killing of Cetuximab-opsonized TNBC cells via ADCC [17]. However, the impact of Cetuximab-mediated ADCC on the activation of DCs has not yet been explored in the BC model.

On the other hand, we have previously shown that NK cells from patients with advanced-stage BC were less efficient in performing ADCC than healthy donors [15]. Exploring approaches to enhance NK cell antitumor capacity, we determined that stimulation with IL-2 or IL-15 cytokines significantly increased NK cell-mediated ADCC [15,17]. IL-15 has emerged as a potential immunotherapeutic candidate for cancer treatment, especially in combination with other agents. IL-15 is structurally related to IL-2 and has overlapping functions, including the promotion of T cell proliferation, cytotoxic T cell differentiation, production of immunoglobulin by B cells, and activation of NK cells [23,24]. In contrast to IL-2, it has no marked effects on Treg cells, does not mediate activation-induced cell death (AICD), and has lower toxicity since it does not cause capillary leak syndrome in mice and non-human primates [23]. Specifically, IL-15 is able to induce NK cell proliferation, survival, and to enhance their cytotoxic functions [25,26]. The effect of the combination of IL-15 and Cetuximab in DC–NK cell interactions has not been studied.

TNBC treatment is still challenging, and immunotherapy is a potential approach in this tumor subtype. Here, we studied whether Cetuximab-mediated ADCC against TNBC cells promoted NK cell activation and DC maturation after the uptake of tumor antigens, which could eventually contribute to the activation of an adaptive antitumor response. Given the immunosuppressive effect of TNBC on NK cells [15], in this work we also tested the combination with IL-15. We show that NK cells activated by TNBC cells opsonized with Cetuximab promoted tumor material uptake and maturation of DCs, as well as their ability to produce IL-12. Furthermore, the stimulation with IL-15 increased the activation of NK cells and the maturation of DCs.

## 2. Materials and Methods

### 2.1. Tumor Cell Lines

The IIB-BR-G cell line was previously established in our laboratory from a primary infiltrating ductal carcinoma [27], and MDA-MB-231 (ATCC^®^ HTB-26^TM^) was acquired from ATCC (Manassas, VA, USA). Both TNBC cell lines harbor K-RAS mutation and overexpress EGFR (Supplementary Figure S1) [17,28]. They were grown in Dulbecco’s modified Eagle’s medium (DMEM; Invitrogen, Carlsbad, CA, USA) supplemented with 10% heat-inactivated fetal bovine serum (FBS, Natocor, Cordoba, Argentina), 2 mM l-glutamine, 10 µg/mL insulin, and 4.5 mg/mL glucose at 37 °C in a humid atmosphere containing 5% CO_2_.

### 2.2. Abs and Reagents

Human rIL-4 and rIL-15 were acquired from PeproTech (Rocky Hill, NJ, USA); human rGM-CSF from Laboratorio Pablo Cassará (Buenos Aires, Argentina). The following mAbs against human molecules were used: FITC or APC-H7 anti-CD3 (SK7), PE anti-CD16 (3G8), APC or BV421 anti-CD56 (NCAM16.2), PE anti-CD25 (M-A251), APC anti-CD69 (FN50), PE or APC anti-CD11c (B-ly6), FITC anti-CD1a (HI149), FITC anti-CD14 (M5E2), FITC anti-CD83 (HB15e), PE anti-CD86 (FUN-1), FITC anti-CD80 (L307.4), PerCP-Cy5.5 anti-HLA-DR (G46-6) and their respective isotype control, all from BD Biosciences (San Jose, CA, USA).

### 2.3. Preparation of Monocyte-Derived DCs and NK Cells from Peripheral Blood Mononuclear Cells (PBMCs)

PBMCs were isolated from buffy coats of healthy donors by density gradient centrifugation using Ficoll-Paque (GE Healthcare, Buckinghamshire, UK). All subjects signed an informed consent approved by the Institutional Review Board of the Instituto Alexander Fleming (Resolution 519/ 1 December 2015). Monocytes were isolated from PBMC using CD14 positive magnetic selection (Miltenyi Biotec, Bergisch Gladbach, Germany) and cultured at 1 × 10^6^ cells/mL for 5 days in RPMI 1640 supplemented with 10% heat-inactivated FBS (New Zeland Origin), 2 mM l-glutamine and 100 U/mL penicillin/streptomycin (complete RPMI, all from Gibco, Carlsbad, CA, USA) with 150 ng/mL GM-CSF and 50 ng/mL IL-4. At day 5, the immature DC population obtained was checked by flow cytometry (>90% CD11c^+^, CD1a^+^, CD14^−^, CD86^low^, and CD83^−^).

Autologous NK cells were isolated by negative magnetic selection (Miltenyi Biotec) from frozen PBMC at day 4 of DC differentiation and rested ON in complete RPMI medium. NK cell purity (>95% CD56^+^ CD3^−^) was assessed by flow cytometry.

### 2.4. Co-Culture of NK Cells, DCs and TNBC Cells

Immature DCs were cultured with TNBC cells and autologous NK cells at a 1:1:1 ratio in the presence of Cetuximab (10 µg/mL, Merck Serono, Darmstadt, Germany) or human myeloma IgG1 isotype control mAb (Sigma-Aldrich, Saint Louis, MO, USA) in complete RPMI medium. When indicated, 10 ng/mL IL-15 was added to the co-cultures. After the stated time, cells were harvested while supernatant was stored at −80 °C until use. Cytokine concentration in 24 h-supernatants was analyzed using ELISA kits for IFN-γ and TNF-α (BD Biosciences) following the manufacturer’s instructions.

### 2.5. Flow Cytometry

The expression of cell surface receptors on DCs and NK cells was analyzed by flow cytometry. After 24 h co-culture, cells were harvested, washed with staining buffer (PBS with 2% FBS), stained using fluorochrome-labeled mAbs for 30 min at 4 °C, and then washed. Cells were acquired in a FACSCanto II flow cytometer (BD) and data were analyzed using FlowJo software (Tree Star, USA). First, we gated cells based on their forward scatter (FSC)–Area versus side scatter (SCC)–Area, and then on single cells using FSC–Area versus FSC–Height dot plots. CD80, CD83, CD86 and HLA-DR expression was evaluated in DC population (CD11c^+^ cells), while in another tube CD25, CD69 and HLA-DR expression was assessed in NK cell population (CD3^−^CD56^+^). In our experimental conditions, NK cells expressed very low levels of CD11c (Supplementary Figure S2), and DCs could be clearly differentiated from NK cells based on FSC vs. SCC plots and CD11c expression. Results were expressed as percentage of positive cells or as normalized geometric mean fluorescence intensity (nMFI): MFI of cells stained with specific mAb/MFI of cells stained with isotype control.

### 2.6. Evaluation of NK Cell-Mediated Cytotoxicity and DC Phagocytosis by Flow Cytometry

**Figure 1:**
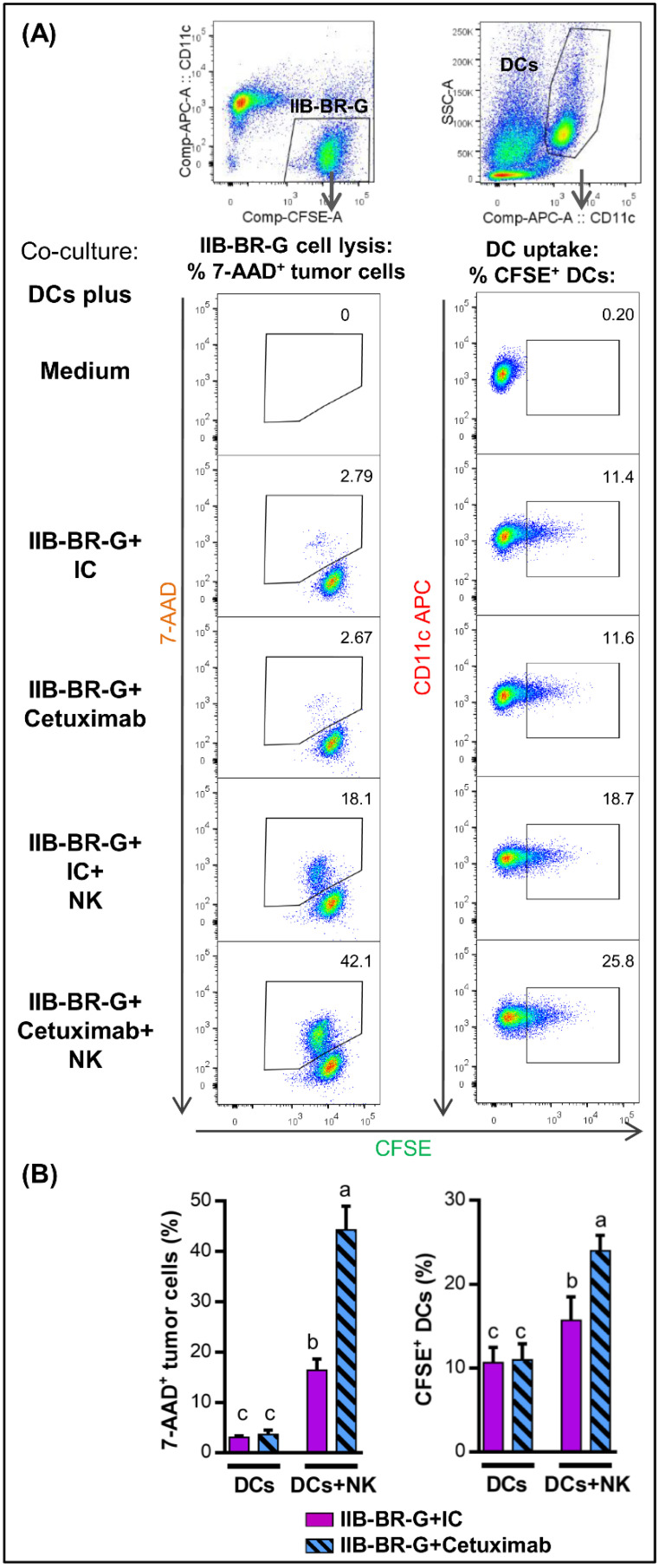
NK cells promoted DC uptake of TNBC cells opsonized with Cetuximab. Cytotoxicity of IIB-BR-G cells (**left**) and their uptake by DCs (**right**) after a co-culture of DCs with IIB-BR-G cells opsonized with 10 µg/mL isotype control (IC) or Cetuximab in the absence (DCs) or presence of NK cells (DCs+NK) at a DCs:NK:IIB-BR-G ratio of 1:1:1 for 4 h at 37 °C. (**A**) Upper panel: simplified gating strategy for CFSE^+^ IIB-BR-G cells (**left**) and CD11c^+^ DCs (**right**). Lower panel: for the different experimental conditions, representative dot plots showing tumor cell lysis (% of 7AAD^+^ cells within CFSE^+^ tumor cells) are presented in the left, and tumor cell uptake by DCs (% of CFSE^+^ cells within the CD11c^+^ DCs) in the right. (**B**) Bars with different letters are statistically different, *p* < 0.05 (ANOVA) (*n* = 3).

To simultaneously evaluate NK cell cytotoxicity and DC phagocytosis of tumor cells by flow cytometry, tumor cells were first labeled with CFSE (Invitrogen) according to the manufacturer’s instructions. After a 4h co-culture, cells were harvested and NK cells were kept unlabeled while DCs were stained with APC anti-CD11c for 30 min at 4 °C. After washing, cells were stained with 7-AAD cell viability stain for 20 min on ice, and immediately acquired. First, single cells were gated as described above. As shown in Figure 1, cytotoxicity was determined based on the percentage of 7-AAD^+^ cells within CFSE^+^ tumor cells. Tumor cell uptake by DCs was evaluated as the percentage of CFSE^+^ cells within the CD11c^+^ population. Parallel to co-cultures at 37 °C, negative controls at 4 °C were carried out.

### 2.7. Interleukin-12p70 Production

After a 24 h co-culture, cell mixture was harvested, washed twice, and plated in 96-well plates at 2 × 10^4^ DCs/well. To mimic the interaction with CD40L-expressing Th cells, CD40L-transfected murine fibroblasts [29] were added at 5 × 10^4^ cells/well for 24 h [30]. Supernatants were analyzed using IL-12p70 ELISA kit (BD Biosciences).

As controls, DCs alone were treated with medium, standard maturation cocktail that induces a phenotype “exhausted” for the production of IL-12 (10 ng/mL IL-1β, 20 ng/mL TNF-α, 50 ng/mL IL-6, 1 µg/mL PGE_2_), and with α1-type polarization cocktail (25 ng/mL IL-1β, 50 ng/mL TNF-α, 1000 UI/mL IFN-γ, 3000 UI/mL IFN-α y 20 µg/mL poly I:C) that induces a polarization with greater capacity to produce IL-12 than immature DCs [30].

### 2.8. Statistical Analysis

GraphPad Prism 7.0 (San Diego, CA, USA) was used for graphs and Infostat 2017 (Cordoba, Argentina) software for the comparison of multiple treatments [31]. ANOVA with randomized block design was performed to analyze data, considering treatments with mAbs, cytokines and their interaction as fixed factors and healthy donors as a random factor (blocks); α = 0.05. Homoscedasticity and normality of residuals were evaluated by visual assessment of plots. If homoscedasticity was not accomplished, models were fitted by the addition of the VarIdent, VarExp, or VarPower variance structure to the random part of the model [32]. Akaike’s and Bayesian’s Information Criteria were used to choose the best variance structure. A *p*-value < 0.05 was considered to be statistically significant.

## 3. Results

### 3.1. NK Cells Promoted DC Uptake of Antigen Material When TNBC Cells Were Coated with Cetuximab

Previously, we demonstrated that a high amount of antigen material is released from TNBC cells as a result of Cetuximab-mediated ADCC [17]. The consequence of this antigen release was investigated in the context of NK cell–DC interaction, using co-cultures including TNBC cells, NK cells and DCs at a 1:1:1 ratio in the presence of 10 µg/mL Cetuximab. NK cell cytotoxicity and DC uptake of tumor cells was assessed in parallel by flow cytometry after a 4 h co-culture at 37 °C. In parallel, negative controls at 4 °C were performed (Supplementary Figure S3).

NK cells increased the lysis of IIB-BR-G cells (% of 7AAD^+^ cells within CFSE^+^ tumor cells), mainly when ADCC was triggered by the opsonization with Cetuximab (Figure 1, left panel). This increase in the percentage of lysis was reflected in an increase in tumor cell uptake by DCs (% of CFSE^+^ cells within the CD11c^+^ DCs), which reached the highest level when both NK cells and Cetuximab were present (Figure 1, right panel). Cetuximab by itself, in the absence of NK cells, did not increase the lysis of the tumor cells nor their uptake by DCs with respect to the isotype control (Figure 1).

### 3.2. IFN-γ and TNF-α Production Increased When NK Cells Were Activated by Cetuximab-Coated TNBC Cells

Next, we studied the activation and production of cytokines by NK cells interacting with DCs when TNBC cells were opsonized with Cetuximab, after a 24 h co-culture.

**Figure 2:**
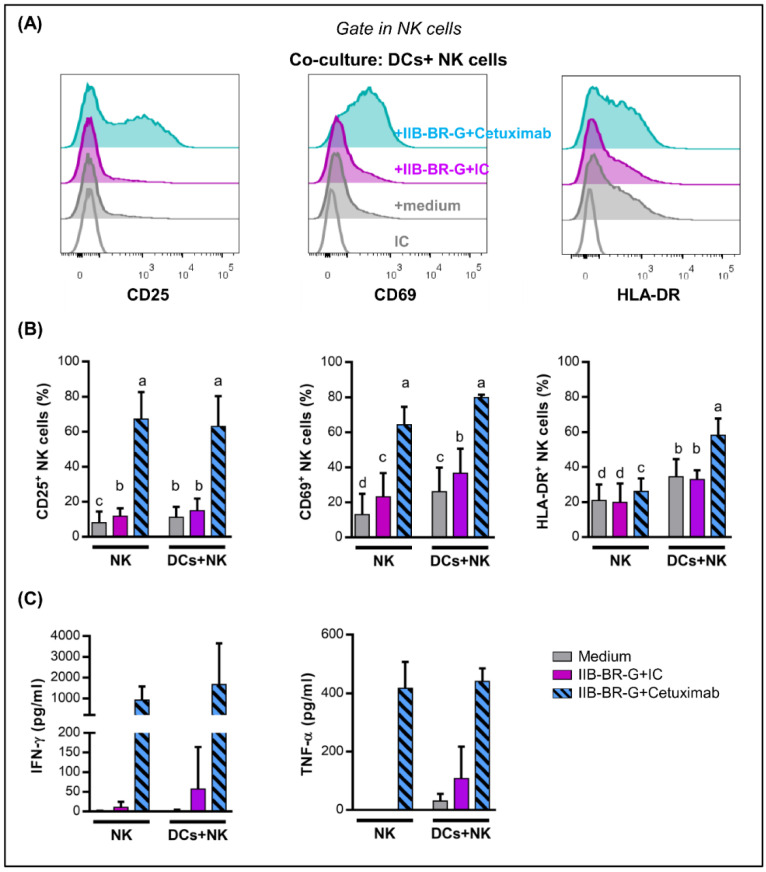
NK cell activation and IFN-γ and TNF-α production were enhanced by Cetuximab opsonization of IIB-BR-G cells. NK cells were co-cultured with IIB-BR-G cells opsonized with IC or Cetuximab, in the absence (NK) or presence of DCs (DCs+NK), in a DCs:NK:IIB-BR-G ratio of 1:1:1 for 24 h. (**A**) Representative histograms showing CD25, CD69 and HLA-DR expression in NK cells after co-cultures in the presence of DCs. (**B**) Expression of CD25, CD69 and HLA-DR in NK cells (*n* = 3–6). Bars with different letters are statistically different, *p* < 0.05 (ANOVA). (**C**) IFN-γ and TNF-α concentration in the co-culture supernatants (*n* = 3–4).

Cetuximab opsonization of IIB-BR-G cells highly increased NK cell expression of CD25, CD69 and HLA-DR (Figure 2A,B), as well as the production of IFN-γ and TNF-α (Figure 2C), compared to isotype control. The expression of these activation markers was similar when co-cultures were performed in the absence of DCs, except for HLA-DR whose induction in NK cells by Cetuximab was higher when DCs were present (Figure 2B).

When NK cells interacting with DCs were co-cultured with MDA-MB-231, another TNBC cell line, Cetuximab opsonization also augmented NK cell activation (Supplementary Figure S4A).

### 3.3. NK Cells Promoted DC Maturation and IL-12 Production When TNBC Cells Were Coated with Cetuximab

Among the diverse activities developed by NK cells, one of outstanding importance is the contribution to DC activation [33,34]. Moreover, activated NK cells can promote IL-12p70 production by DCs. IL-12p70 is a critical cytokine for the induction of Th1 cells, which are considered to be needed for optimal cancer treatment [35].

**Figure 3:**
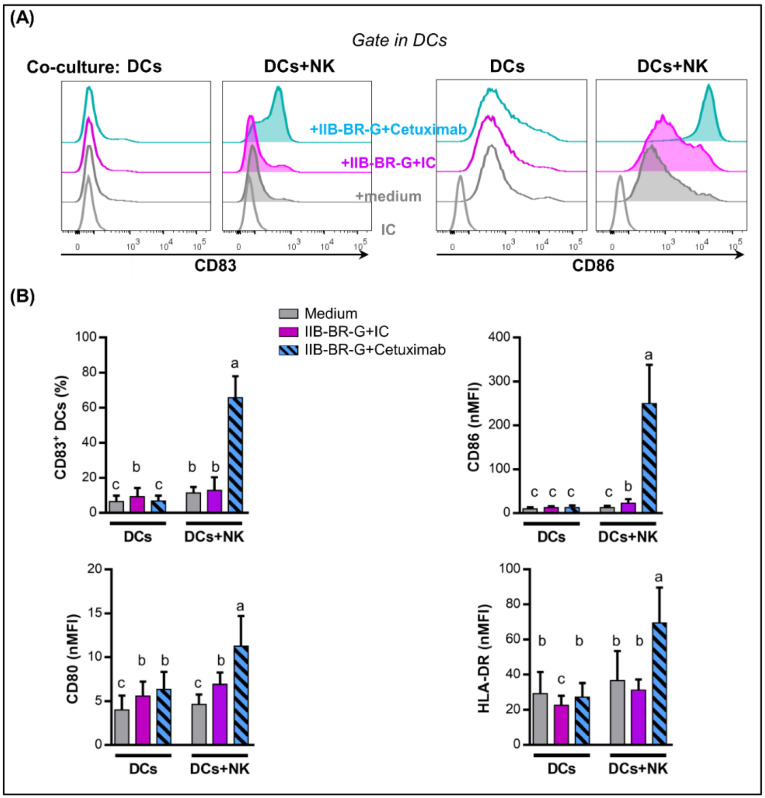
NK cells promoted DC maturation when IIB-BR-G cells were coated with Cetuximab. DCs were co-cultured with IIB-BR-G opsonized with IC or Cetuximab, in the absence (DCs) or presence of NK cells (DCs+NK), in a DCs:NK:IIB-BR-G ratio of 1:1:1 for 24 h. (**A**) Representative histograms showing CD83 and CD86 expression in DCs after co-cultures in the presence of NK cells. (**B**) Expression of CD83, CD86, CD80 and HLA-DR in DCs. Bars with different letters are statistically different, *p* < 0.05 (ANOVA) (*n* = 3–6). nMFI: normalized geometric mean fluorescence intensity.

Next, we studied DC maturation after a 24-h co-culture. As shown in Figure 3, the expression of CD83, CD86, CD80 and HLA-DR in DCs increased when IIB-BR-G cells were opsonized by Cetuximab compared to isotype control only when NK cells were present to mediate ADCC and cytokine production. In contrast, Cetuximab did not affect DC maturation in the absence of NK cells (Figure 3) or tumor cells (Supplementary Figure S5).

These results were replicated using another TNBC cell line: DC maturation increased when MDA-MB-231 cells were opsonized by Cetuximab in the presence of NK cells (Supplementary Figure S4B).

**Figure 4:**
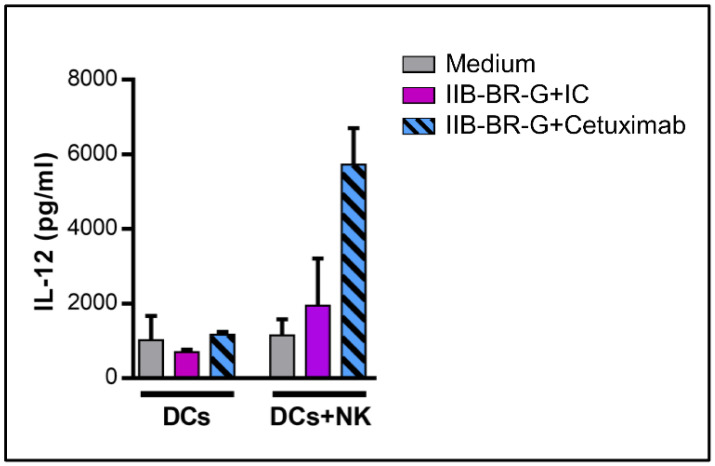
NK cells promoted IL-12 production by DCs when TNBC cells were coated with Cetuximab. IL-12p70 concentration in the 24-h co-culture supernatant of cells transfected with CD40L and DCs, which were first stimulated with IIB-BR-G cells opsonized with IC or Cetuximab, in the absence (DCs) or presence of NK cells (DCs+NK) for 24 h (*n* = 2).

To investigate the capacity of DCs maturated in the previous conditions to produce IL-12p70, cells were harvested, washed and incubated with a cell line expressing CD40L to mimic the interaction with CD4 T cells [30]. DCs that were co-cultured with NK cells activated by TNBC cells opsonized with Cetuximab had an increased ability to produce IL-12p70 after subsequent CD40L stimulation compared to the opsonization with the isotype control. In contrast, IL-12 production did not improve under any circumstances when NK cells were absent during co-culture (Figure 4).

To sum up, NK cells activated by Cetuximab-opsonized TNBC cells promoted DC maturation and IL-12 production.

### 3.4. IL-15 Increased NK Cell Activation and DC Maturation Triggered by Cetuximab

Previously, we demonstrated that IL-15 increases ADCC and cytokine production by NK cells against TNBC cells opsonized by Cetuximab [17]. Therefore, we investigated IL-15 effect on NK cell activation and DC maturation by adding 10 ng/mL of IL-15 to the 24 h co-cultures.

**Figure 5:**
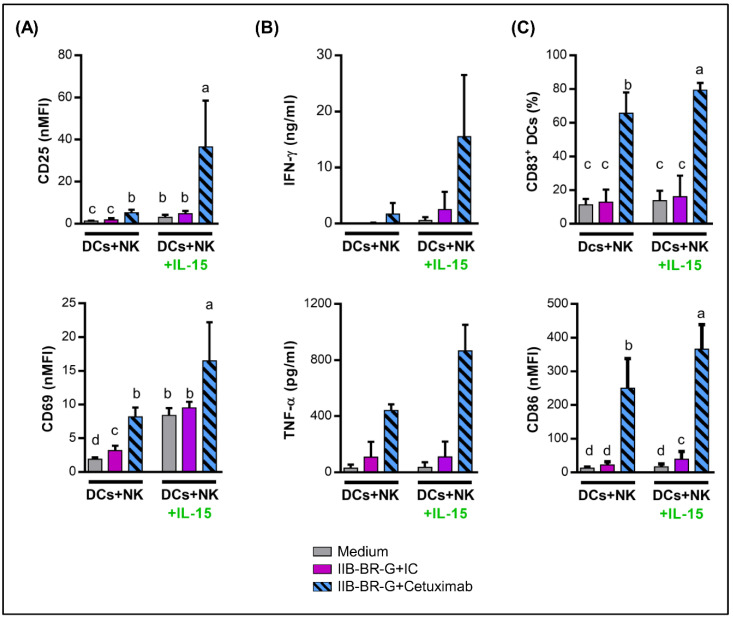
IL-15 increased NK cell activation and DC maturation triggered by Cetuximab. DCs were co-cultured with NK cells and IIB-BR-G cells opsonized with IC or Cetuximab in the absence (DCs+NK) or presence of 10 ng/mL IL-15 (DCs+NK+IL-15) for 24 h. (**A**) Expression of CD25 and CD69 in NK cells (*n* = 4). (**B**) Concentration of IFN-γ and TNF-α in the co-culture supernatant (*n* = 3–4). (**C**) Expression of CD83 and CD86 in DCs (*n* = 6). Bars with different letters are statistically different, *p* < 0.05 (ANOVA).

In concordance with our previous results, stimulation with IL-15 augmented NK cell activation triggered by Cetuximab-opsonized cells, showing an increase in the expression of CD25 and CD69 (Figure 5A) and higher release of IFN-γ and TNF-α (Figure 5B) compared to control without IL-15.

In parallel, IL-15 augmented DC maturation induced by Cetuximab-activated NK cells, evidenced as an increase in the expression of CD83 and CD86 in DCs compared to the control without stimulation (Figure 5C). In contrast, IL-15 did not affect DC maturation in the absence of NK cells (Supplementary Figure S6).

## 4. Discussion

Treatment options for TNBC are still limited. Chemotherapy is currently the main systemic treatment and, although patients tend to respond initially to this therapy, they recur more frequently than other breast cancer subtypes [2]. Targeting EGFR with humanized mAb Cetuximab is used for the treatment of various cancers, and preclinical and translational research suggests that a considerable proportion of TNBC are dependent on EGFR signaling [36].

Previously, we determined that Cetuximab promotes NK cell activation against TNBC cells through ADCC [17]. Preclinical and clinical evidence supports that antitumor activity mediated by innate cells, such as ADCC, could contribute to the short-term therapeutic response to mAbs directed against tumor-associated antigens, while long-term clinical efficacy would be based on the generation of an adaptive antitumor immune response, for which the participation of antigen-presenting cells is necessary [37,38]. In a murine model using a HER-2+ breast carcinoma cell line transfected with EGFR, Cetuximab-mediated tumor regression depended on innate and adaptive immune responses. In fact, the innate MyD88 signaling pathway and FcγR were involved in therapeutic effect of Cetuximab, and CD8^+^ T cell depletion diminished Cetuximab mediated tumor regression [39]. Therefore, here, we studied how NK cells interact with DCs after activation by ADCC. For this, we set up a human in vitro model in which NK cells, DCs and TNBC cells were co-cultured in the presence of Cetuximab.

First, in the absence of NK cells, the addition of Cetuximab did not affect the lysis of IIB-BR-G cells or their uptake by DCs. The first is due to the fact that the cells lines used in this study are resistant to the antiproliferative and proapoptotic mechanisms of Cetuximab because they present a mutation in K-RAS [17,28]. Mutations in K-RAS are a rare oncogenic event in TNBC [40,41], but more frequent in cell lines established from them. However, we show that the addition of NK cells increased the lysis of IIB-BR-G cells and their uptake by DCs, mainly when ADCC was triggered by the addition of Cetuximab.

Next, we studied whether the opsonization of TNBC cells with Cetuximab could improve the maturation of DCs. In the absence of NK cells, Cetuximab did not affect DC maturation. Circulating immune complexes and cell-bound Ig represent a potential stimulus for DC activation. However, they express both activating and inhibitory Fcγ receptors (FcγRIIA and FcγRIIB, respectively), and the result of their stimulation depends on the degree of expression of each of these receptors and their affinity for the Fcγ region of the mAb under study [42]. Therefore, blocking inhibitory receptors may be necessary for Cetuximab to activate DCs, as was observed in a glioma model [43]. In contrast, in the presence of NK cells, opsonization with Cetuximab significantly increased the maturation of DCs, with an increase in the expression of CD83 activation marker, CD80 and CD86 co-stimulatory molecules, and HLA-DR antigen presenting molecule. Accordingly, in a Head and Neck Cancer (HNC) model, the maturation of DCs was promoted by Cetuximab-activated NK cells in the presence of tumor cells, and this effect was partially dependent on the IFN-γ produced by NK cells [44].

Not only are NK cells capable of activating DCs, but reciprocally, DCs can stimulate NK cells. When studying the activation of NK cells in these co-cultures, we show that the presence of TNBC cells opsonized with Cetuximab was a strong stimulus for their activation, increasing their expression of CD69 and CD25 (IL-2Rα), and the production of IFN-γ and TNF-α. However, these activation markers were similar both in the presence and absence of DCs. This differs from that observed in the HNC model, in which DCs reciprocally stimulated NK cells, showing a significant increase in IFN-γ production when DCs were added to the co-culture of NK cells and HNC cells opsonized with Cetuximab. Moreover, we studied the expression of HLA-DR, which is used as another NK cell activation marker since its expression increases after stimulation with IL-2, and could be involved in the presentation of antigens to CD4^+^ T lymphocytes [10,45,46]. In contrast to other activation markers, the increase in HLA-DR expression in NK cells triggered by Cetuximab was higher when DCs were present in the co-culture.

IL-12 produced by DCs after activation plays an essential role in cross-talk with NK cells and the polarization of the immune response towards a Th1 profile. In our model, after stimulation of DCs with NK cells activated by Cetuximab, IL-12 levels in co-culture supernatants were undetectable (result not shown). This is in line with previous evidence showing that IFN-γ produced by NK cells does not stimulate the production of IL-12 by itself, but primes DCs for IL-12 production after another stimulus, such as the interaction with CD40L on the surface of Th CD4^+^ lymphocytes [47]. We demonstrated that NK cells activated by Cetuximab-opsonized IIB-BR-G cells promoted the production of IL-12 by DCs after stimulation with CD40L. Finally, these DCs could cross-present tumor antigens to CD8^+^ T cells. In this sense, Deauvieau et al. demonstrated that NK cells activated by tumor cells opsonized with IgG1 therapeutic mAbs increased cross-presentation of tumor antigens to CD8^+^ T lymphocytes by DCs, which was dependent on soluble factors (IFN-γ and TNF-α). Although, in this case, NK cells did not increase the uptake of tumor material by DCs, their presence was necessary during this process, indicating that they could promote a specific pathway of capture or antigen processing suitable for cross-presentation [48].

Cetuximab has been tested in several phase I-II studies in TNBC with only modest benefit [22,49,50,51]. Because a small portion of patients demonstrate a response to EGFR inhibitors, it may be necessary to stratify patients to enhance the efficacy of these therapies and to develop effective combination strategies for this patient population. In a pilot Phase II neoadjuvant trial testing Cetuximab combined with docetaxel, the pre-therapy ratio between CD8^+^ and FOXP3^+^ TILs was predictive of pathologic complete response, showing that the immune component of the tumor microenvironment may play an important role in predicting TNBC response to this neoadjuvant therapy [50]. Next, we proposed that the joint activation of the immune system by the combination of mAbs and cytokines could contribute to the therapeutics of this tumor. This is a potential approach to overcome the immunosuppressive activity of tumor cells on patient’s NK cells. Since we previously demonstrated that IL-15 increases the production of IFN-γ and TNF-α by NK cells triggered by Cetuximab [17], we studied its effect on NK cell–DC interaction.

Here, we showed that IL-15 promoted a significant increase in the activation of NK cells against TNBC cells opsonized with Cetuximab, which increased their expression of CD69 and CD25, and their production of IFN-γ and TNF-α. Consequently, IL-15 increased the maturation of DCs in the presence of NK cells activated by Cetuximab. Recently, phase I clinical trials have tested human recombinant IL-15 (rhIL-15) as intravenous bolus monotherapy [52], subcutaneous injection [53] and continuous intravenous infusion [54]. The latter induced a massive 38-fold increase in total circulating NK cells and 358-fold increase in CD56^bright^ NK cells. Adverse effects typically related to the administration of recombinant cytokines were observed. Currently, IL-15 is being tested in combination with other mAbs directed against tumor-associated antigens, with Alemtuzumab (anti-CD52) in adult patients with T-cell leukemia (NCT02689453) and Obinutuzumab (anti-CD20) in patients with chronic lymphocyte leukemia (NCT03759184). Furthermore, different strategies have been developed to increase the in vivo half-life and efficacy of IL-15, mainly by generating IL-15/IL-15Rα conjugates. These new agents renew the prospect of IL-15 as a cancer immunotherapeutic agent [55].

## 5. Conclusions

In this work, we show that NK cells activated by TNBC cells opsonized with Cetuximab promote tumor material uptake and maturation of DCs, as well as their ability to produce IL-12. Furthermore, the immunostimulatory cytokine IL-15 increased the activation of NK cells and the maturation of DCs. These results were the first to demonstrate the effects of an IgG1 therapeutic mAb on the interaction between NK cells and DCs in a TNBC model and, additionally, that these effects can be improved with IL-15.

## Supplementary Materials

The following are available online at https://www.mdpi.com/2073-4409/9/7/1573/s1, Figure S1: EGFR expression in TNBC cell lines used in this study; Figure S2: Expression of CD11 in DCs and NK cells; Figure S3: Cytotoxicity of IIB-BR-G cells and their uptake by DCs at 4 °C; Figure S4: NK cell activation and DC maturation against MDA-MD-231 cells coated with Cetuximab; Figure S5: Expression of CD83 in controls; Figure S6: Effect of IL-15 in the absence of NK cells.
