# Supplementary material for: Cetuximab and IL-15 Promote NK and Dendritic Cell Activation In Vitro in Triple Negative Breast Cancer
Source: Cells. 2020 Jun 28;9(7):1573. doi: 10.3390/cells9071573 (PMC7408037; doi:10.3390/cells9071573)
Supplement: Supplementary file 1 [file cells-09-01573-s001.pdf]

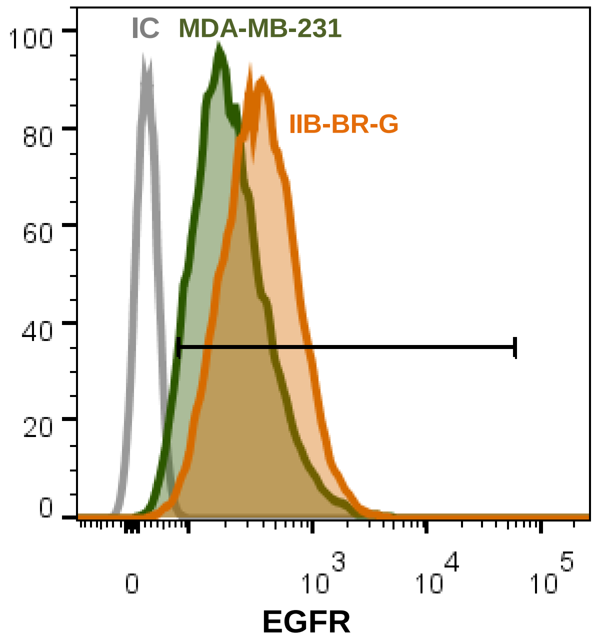

Supplementary Figure 1. EGFR expression in TNBC cell lines used in this study. EGFR expression in IIB-BR-G and MDA-MB-231 cell lines was evaluated by flow cytometry, after incubating cells with Cetuximab during 30min at 4ºC, washing, and staining with polyclonal rabbit anti-human IgA/IgG/IgM/Kappa/Lambda conjugated with FITC (Dako) for 30min at 4ºC.

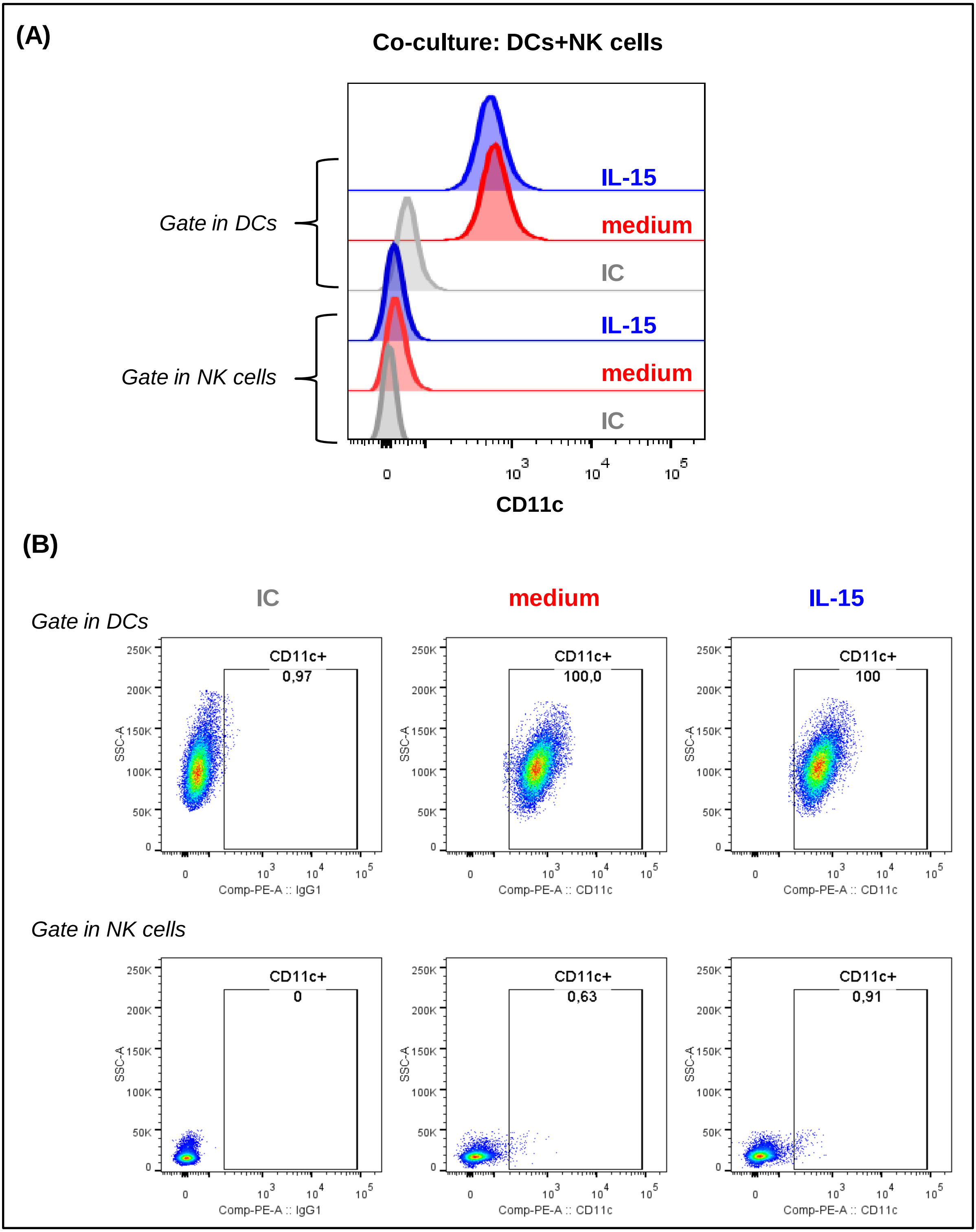

Supplementary Figure 2. Expression of CD11 in DCs and NK cells. DCs and NK cells were co-cultured in the absence or presence of 10 ng/ml IL-15 for 24hs.

(A) Representative histograms showing the expression of CD11c on both cell types.

(B) Representative pseudocolor graphs showing the expression of CD11c on both cell types.

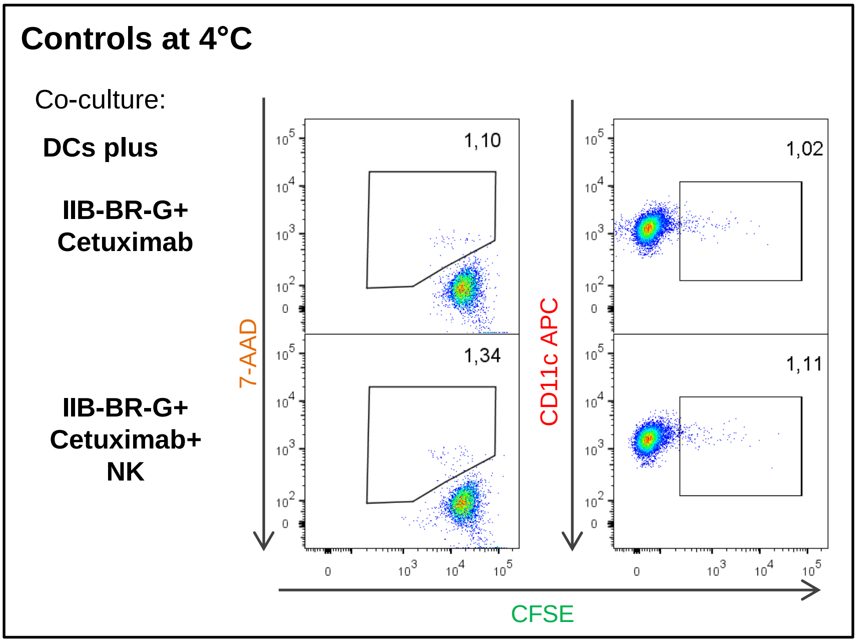

Supplementary Figure 3. Cytotoxicity of IIB-BR-G cells and their uptake by DCs after a co-culture of DCs with IIB-BR-G plus Cetuximab, in the absence or presence of NK cells, for 4 hours at 4°C. Representative graphs showing one of three experiments carried out independently.

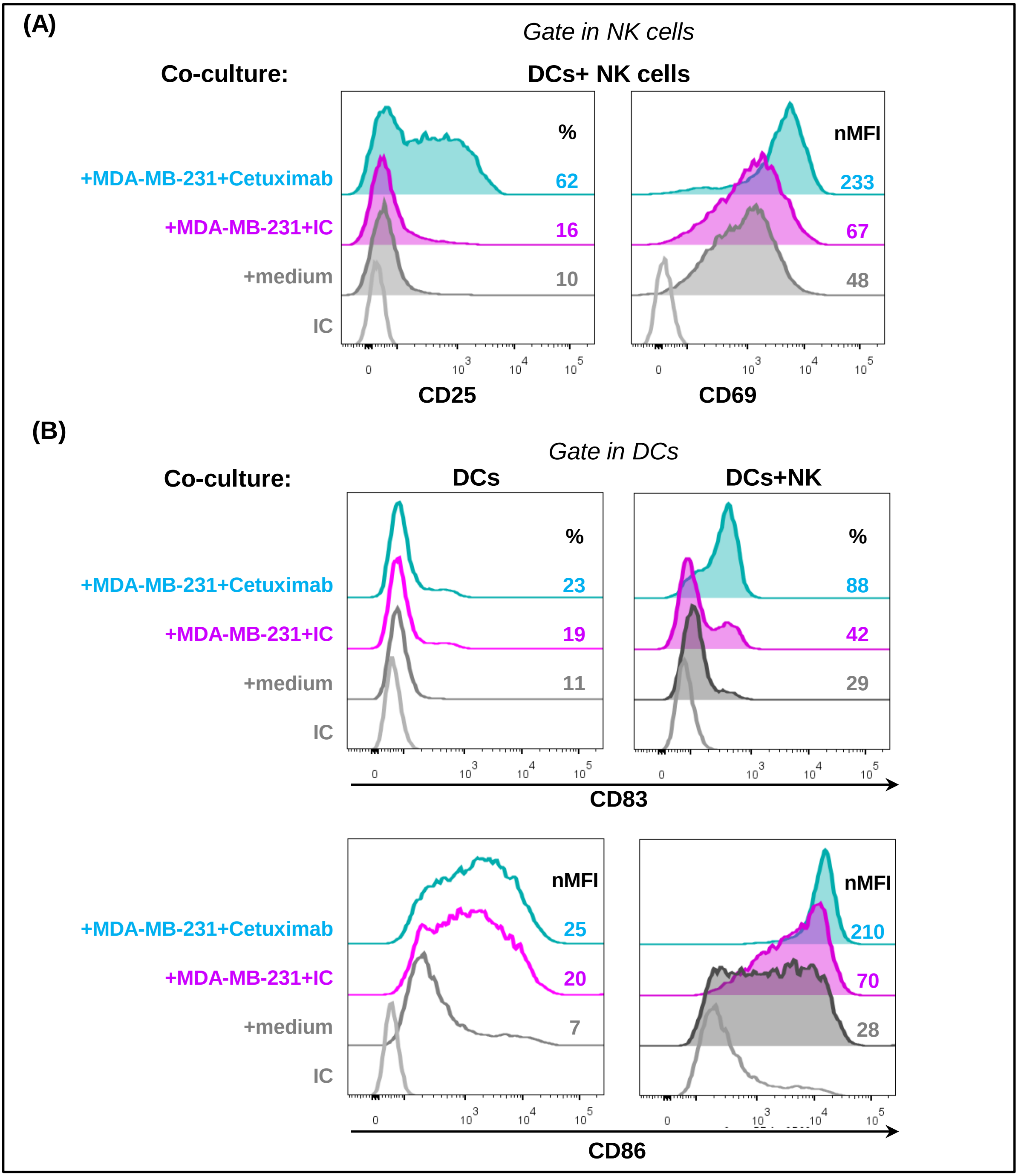

Supplementary Figure 4. NK cell activation and DC maturation against MDA-MD-231 cells coated with Cetuximab. DCs were co-cultured with MDA-MD-231 cells opsonized with IC or Cetuximab, in the absence or presence of NK cells, in a DCs:NK:MDA-MD-231 ratio of 1:1:1 for 24h. Representative histograms showing one of two experiments performed independently. %: percentage of positive cells, nMFI: normalized geometric mean fluorescence intensity.

(A) NK cell activation was enhanced by Cetuximab opsonization of MDA-MD-231 cells.

(B) NK cells promoted DC maturation when MDA-MD-231 cells were coated with Cetuximab.

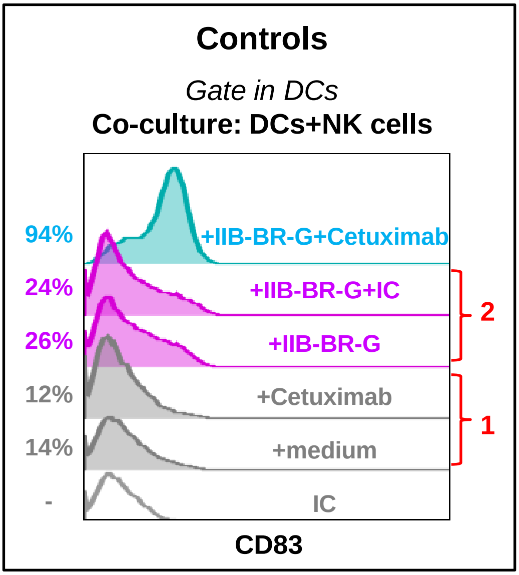

Supplementary Figure 5. Expression of CD83 in controls. DCs co-cultured with NK cells, with the addition of medium or Cetuximab (Key 1), IIB-BR-G with medium or opsonized with IC (Key 2) or Cetuximab, during 24 hours. Representative histograms showing one of two experiments performed independently.

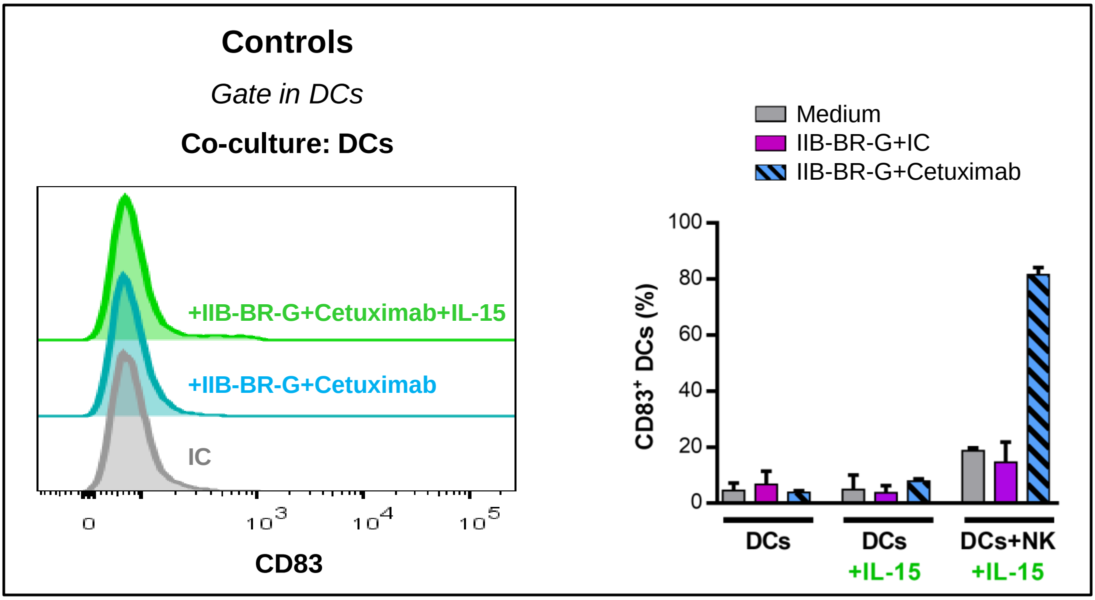

Supplementary Figure 6. Effect of IL-15 in the absence of NK cells. Expression of CD83 in DCs after co-culture with IIB-BR-G cells opsonized with Cetuximab without NK cells, in the absence (DCs) or presence of 10 ng/ml IL-15 (DCs+IL-15) for 24 hours. (A) Representative histograms of one of two experiments performed independently. (B) As control, co-culture in the presence of NK cells and IL-15 (DC+NK+IL-15) is shown (n= 2).
